# Impact of Lifestyle Intervention on HDL-Induced eNOS Activation and Cholesterol Efflux Capacity in Obese Adolescent

**DOI:** 10.1155/2016/2820432

**Published:** 2016-11-14

**Authors:** Jenny Wesnigk, Luc Bruyndonckx, Vicky Y. Hoymans, Ann De Guchtenaere, Tina Fischer, Gerhard Schuler, Christiaan J. Vrints, Volker Adams

**Affiliations:** ^1^University of Leipzig, Heart Center Leipzig, Leipzig, Germany; ^2^Antwerp University Hospital, Laboratory of Cellular and Molecular Cardiology, Edegem, Belgium; ^3^Antwerp University Hospital, Department of Pediatrics, Edegem, Belgium; ^4^Cardiovascular Diseases, Department of Translational Pathophysiological Research, University of Antwerp, Antwerp, Belgium; ^5^Laboratory of Experimental Medicine and Pediatrics, University of Antwerp, Antwerp, Germany; ^6^Zeepreventorium, De Haan, Belgium

## Abstract

*Background.* Endothelial dysfunction occurs in obese children and adolescent and is regarded as a key step in the development of atherosclerosis. Important components for the development of endothelial dysfunction are reduced activity of endothelial nitric oxide synthase (eNOS) and an increase in cholesterol deposition in the vessel wall, due to reduced reverse cholesterol transport (RCT) activity. High density lipoprotein (HDL) exhibits antiatherosclerotic properties including modulation of eNOS activity and cholesterol efflux capacity. Lifestyle intervention programs can modify endothelial dysfunction in obese adolescents, but their impact on HDL-mediated eNOS activation and RCT is unknown so far.* Methods.* Obese adolescents (15 ± 1 years, BMI > 35 kg/m^2^) where randomized either to an intervention group (IG, *n* = 8; restricted diet and exercise) or to a usual care group (UC, *n* = 8). At the beginning and after 10 months of treatment HDL-mediated eNOS phosphorylation and cholesterol efflux capacity were evaluated.* Results.* Ten months of treatment resulted in a substantial weight loss (−31%), an improvement of endothelial function, and an increase in HDL-mediated eNOS-Ser^1177^ phosphorylation and RCT. A correlation between change in eNOS-Ser^1177^ phosphorylation or RCT and change in endothelial function was noted.* Conclusion.* A structured lifestyle intervention program improves antiatherosclerotic HDL functions, thereby positively influencing endothelial function.

## 1. Introduction

Adolescent obesity is rising at an alarming rate. The worldwide prevalence of childhood overweight/obesity increased from 4.2% in 1990 to 6.7% in 2010, and it is predicted to reach 9.1% in 2020 [1]. Obesity is closely related to multiple diseases such as type two diabetes mellitus, coronary artery disease, hypertension, dyslipidemia, and fatty liver disease [2] and is a predictor of an increased risk for cardiovascular disease and its related mortality [3, 4]. Endothelial function is impaired in up to 50% of prepubertal and pubertal obese children [5, 6]. The most important factor for regulating endothelium-dependent vasodilation is the bioavailability of nitric oxide (NO), which depends on its synthesis by endothelial nitric oxide synthase (eNOS) and degradation by reactive oxygen species (ROS). The activity of eNOS can be regulated by modulating its RNA/protein expression or by phosphorylation of the enzyme at specific sites, like serin-1177 and threonine-495 [7].

High density lipoprotein (HDL) is a complex organized particle which inversely relates to the risk of myocardial infarction and even death [8]. Unfortunately, all efforts to increase HDL concentration by pharmaceutical interventions in adults failed to result in changes in cardiovascular risk or recurrent cardiovascular events [9]. Therefore, in recent years the functional aspect of HDL, and not only the concentration, has become more and more important [10]. Besides promoting reverse cholesterol transport and its anti-inflammatory action [11], recent studies documented that HDL also regulates NO bioavailability by modulating eNOS enzymatic activity [12–14]. This ability to activate eNOS via phosphorylation is impaired in diabetes [15], heart failure [12], and obesity [16].

Since pharmaceutical and surgical treatment option are very limited to fight childhood and adolescent obesity, lifestyle interventions involving nutrition and physical activity are crucial. In a recent clinical study we could show that an intervention combining supervised diet and exercise training decreased BMI and body fat significantly and resulted in an improved endothelial function when compared to a usual care group [17]. Whether this improvement in endothelial function is accompanied by an improvement in HDL function (eNOS phosphorylation and cholesterol efflux) remains unclear. In a small cohort of obese children a defined 6-month lifestyle intervention resulted in no change in HDL-mediated phosphorylation of eNOS [16]. It is important to note that the lifestyle intervention in that specific study was not sufficiently effective in reducing the body weight so that the children were still classified as obese at the end of the program. A study in obese women showed a strong correlation between the change in body weight and the change in cholesterol efflux capacity but also noted that a substantial weight loss is necessary to improve reverse cholesterol transport [18].

To critically assess if lifestyle intervention can modify HDL function in obese adolescents, we assessed HDL-mediated eNOS phosphorylation and RCT in obese adolescents participating in a 10-month lifestyle intervention program which empowered the participants to lose a substantial amount of weight.

## 2. Methods

### 2.1. Patient Population, Blood Sampling, and Endothelial Function

The patients analyzed in the present study are a subgroup of the patients recently engaged in a trial investigating the impact of diet and exercise on endothelial function in obese adolescents [17]. Eight adolescents from the intervention group and 8 individuals from the usual care group were selected. Groups were matched for age, sex, pubertal development, BMI, and physical activity. Blood was taken before randomization (lifestyle intervention or usual care) at study begin and after 10 months. Serum was isolated by centrifugation and stored at −80°C until analyzed. Endothelial function was measured with pulse amplitude tonometry [17].

### 2.2. Lifestyle Intervention Program

A detailed description of the intervention program is described in our recent publication [17]. In general the intervention program (10-month duration) included dietary restriction (1500–1800 kcal/day), supervised physical activity, and psychological support.

Adolescents in the usual care group were treated by their general pediatrician, focusing on caloric restriction and encouragement to participate in sports activities.

### 2.3. HDL Isolation

HDL was isolated by sequential ultracentrifugation according to the method originally described by Havel et al. using solid potassium bromide for density adjustment [19]. In brief, the density of serum was raised to 1.006 by the addition of potassium bromide (KBr) and subjected to ultracentrifugation (24 h, 4°C, and 50,000 rpm, TV-865 rotor). After centrifugation, the lower half was transferred to a new tube and the density was adjusted to 1.036 with KBr. After centrifugation (same conditions as above) the lower half of this second step was transferred again to a new tube and the density was adjusted to 1.21 with KBr. The tube was subjected again to centrifugation (same conditions as above). After this last centrifugation the HDL (yellow band) in the upper half of the tube was collected. The quality of isolated HDL was evaluated by polyacrylamide gel electrophoresis followed by Coomassie Brilliant Blue staining.

### 2.4. Cell Culture Experiments and Western Blot Analysis

Human aortic ECs (HAEC; Cell Systems Biotechnology, Troisdorf, Germany) were cultured in EGM-2 cell culture medium (Lonza, Walkersville, MD) and incubated for 0, 5, 10, 15, 30, or 60 minutes with 50 *μ*g/mL isolated HDL. Thereafter, cells were harvested with ice-cold lysis buffer (50 mmol/L Tris-HCl; pH 7.4; 1% NP-40; 0.25% Na-deoxycholate; 150 mmol/L NaCl; 1 mmol/L EDTA; 0.1% Triton X-100; 0.2% SDS) containing protease inhibitor mix M (Serva, Heidelberg, Germany) as well as phosphatase inhibitor mix II (Serva). Protein concentration was determined using BSA as standard (BCA method; Pierce, Rockford, IL).

Ten micrograms of total protein was separated on a denaturing polyacrylamide gel and transferred to a PVDF membrane. To detect specific proteins, the following antibodies were applied: anti-eNOS (Santa Cruz), antiphospho-eNOS-Ser1177, antiphospho-eNOS-Thr495 (both BD Biosciences, Heidelberg, Germany). For the evaluation of HDL-induced phosphorylation of eNOS, the maximal stimulation was used as recently described [12, 16]. All samples were analyzed in triplicate.

### 2.5. Cholesterol Efflux Assay

Cholesterol efflux, mediated by the ATP-binding cassette transporter A1 (ABCA1), was measured in duplicate using ^3^[H]-cholesterol (Perkin Elmer, Boston, MA, USA) labelled J744 macrophages and apolipoprotein B- (ApoB-) depleted serum as recently described [20]. In brief, ApoB depleted serum was generated by mixing 100 *μ*L patient serum with 40 *μ*L of polyethylene glycol (PEG, 40% in glycine buffer pH 7.4). After incubating the mixture for 20 minutes at room temperature and centrifugation (30 min, 10,000 ×g, 4°C) the supernatant (ApoB depleted serum) was collected and frozen at −80°C until used in the cholesterol efflux assay. J774 macrophages were grown overnight at a density of 300,000/cm^2^ in DMEM (Gibco) supplemented with 10% FCS and Penicillin/Streptomycin (Pen/Strep, Sigma). The following day medium was replaced by DMEM supplemented with 10% FCS, Pen/Strep, 2 *μ*g/mL Sandoz 58-035 (Sigma), 0.3 mM 8-(4-chlorophenylthio)cyclic-AMP (Sigma), and 1 *μ*Ci/mL ^3^[H]-cholesterol. The next morning the medium was removed and the cells were incubated for 2 h with DMEM (serum free), Pen/Strep and 0.2% BSA fat free to remove cholesterol not incorporated into the cell membrane. Subsequently, efflux medium (DMEM serum free) containing 2.8% apolipoprotein B-depleted serum was added to the cells and incubated for 3 h. Liquid scintillation counting of the cell supernatant and the lysed cells was used to calculate the % efflux capacity.

### 2.6. Statistical Analyses

Data are presented as mean ± SD. Normal distribution of the data was tested (Kolmogorov and Smirnov test), and between-group differences were assessed by paired or unpaired *t*-test. Significance was accepted as *p* < 0.05. Analyses were performed by SPSS version 22 (SPSS Inc., Chicago, USA).

## 3. Results

### 3.1. Patient Characteristics

At study beginning there was no significant difference in baseline characteristics of the individuals randomized either to the control or to intervention group (Table 1). All study participants were severely overweight with a BMI above 35. In the intervention group, 10 months of lifestyle intervention resulted in a 32% reduction in BMI (*p* < 0.001), a 41% reduction in body fat (*p* < 0.001), and a 10% increase in maximal oxygen uptake (*p* = 0.002). Endothelial function showed a trend towards improvement but did not reach statistical significance (max. dilation *p* = 0.072), whereas the pulse wave velocity, a marker for the vessel stiffness, decreased significantly in the intervention group. In the usual care group no changes in the aforementioned parameters were detected after 10 months.

### 3.2. HDL-Mediated eNOS Phosphorylation

**Figure 1:**
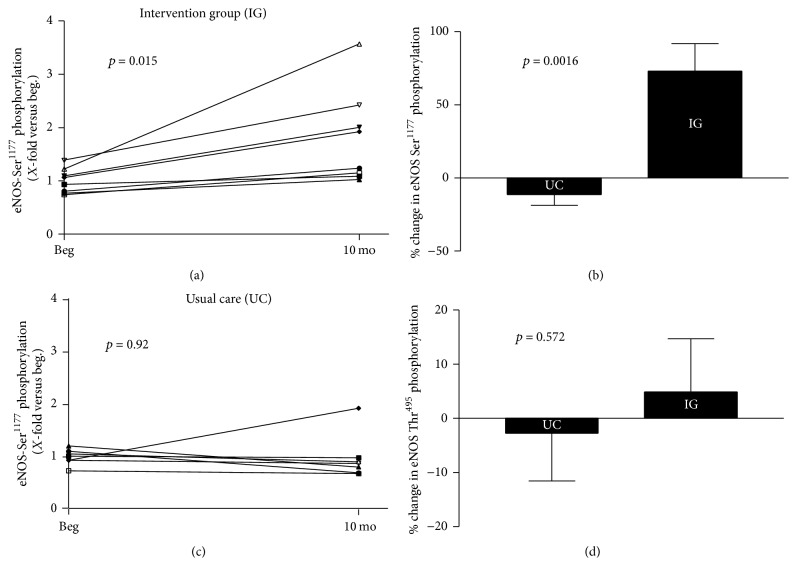
Impact of lifestyle intervention program on HDL-mediated eNOS phosphorylation. Ten months of intervention increases HDL-mediated eNOS-Ser1177 phosphorylation in the intervention group when analyzed over time (a) or compared to usual care group (UC) (b). No change in HDL-mediated eNOS-Ser^1177^ phosphorylation occurs in UC (c). Phosphorylation of the eNOS-Thr^495^ residue was not affected by the intervention program when compared to UC (d).

The HDL-mediated phosphorylation at position Ser^1177^ of eNOS significantly improved in the intervention group (Figure 1(a)). Calculating the % change over the intervention period of 10 months, a significant difference between the intervention group and the usual care was detected (Figure 1(b)). In the intervention group a 73% increase in Ser^1177^ phosphorylation was evident. In the usual care group no difference in eNOS-Ser1177 phosphorylation was found at start versus study end (Figure 1(c)). There was no significant difference in % change of HDL-mediated eNOS phosphorylation at Thr^495^ (Figure 1(d)). A correlation (*r* = 0.54, *p* = 0.046) was evident between changes in eNOS-Ser^1177^ phosphorylation and the change in endothelial function, measured as max. dilation.

### 3.3. Reverse Cholesterol Efflux

**Figure 2:**
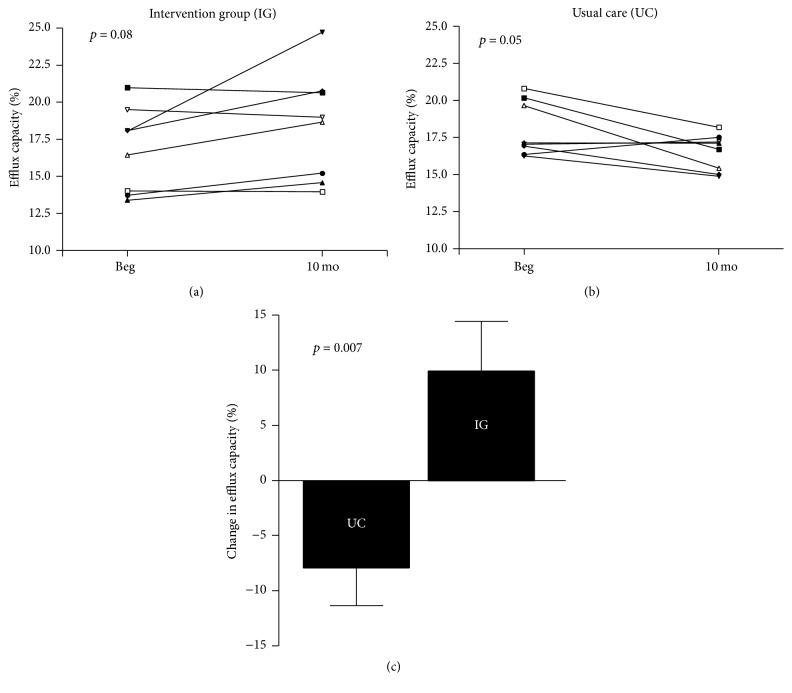
Impact of lifestyle intervention program on cholesterol efflux capacity. Individual changes were observed in the intervention group (IG) (a) and the usual care group (UC) (b). Comparing the change in cholesterol efflux capacity between IG and UC group (c) a significant difference was obvious.

When analyzing the efflux capacity of HDL from adolescents in the usual care and intervention group, at 10 months a decline (*p* = 0.05) was observed in the usual care group (Figure 2(a)), whereas an increase (*p* = 0.08) was noted in the intervention group (Figure 2(b)). Accordingly, the change in HDL efflux capacity from study beginning to end was significantly higher in the adolescents from the intervention group than those who received usual care (Figure 2(c)). There was a moderate correlation (*r* = 0.56, *p* = 0.035) between the changes in efflux capacity and the change in endothelial function, measured as max. dilation.

## 4. Discussion

Obesity and low physical activity have negative effects on endothelial function, whereas lifestyle interventions can attenuate or even reverse this process. One factor able to influence endothelial function is HDL via its modulation of reverse cholesterol transport and activation of eNOS. Several findings emerge from the present study.

First, the phosphorylation of eNOS at the activating site Ser^1177^ was improved by a 10-month intervention program which combined dietary restriction, physical activity, and psychological support. No change in HDL-mediated phosphorylation at the Thr^495^ residue was shown. Second, the decline in HDL-mediated cholesterol efflux was increased by the intervention program.

Third, correlations between the change in eNOS phosphorylation at Ser^1177^ or the efflux capacity and the change in endothelial function were observed. These correlations support the importance of the changes in HDL function for influencing endothelial function.

Taken together, the results of the present study documented that an intervention program, able to achieve significant weight loss, has beneficial effects on HDL function (eNOS activation and cholesterol efflux). This effect on HDL function may be one way how lifestyle intervention is able to improve endothelial function and reduces the risk of developing atherosclerosis.

### 4.1. Lifestyle Intervention Program and Impact on HDL-Mediated eNOS Activation

Studies have shown that HDL, acting via the SR-B1 receptor, can activate eNOS to generate NO [21, 22] and induce vasodilation [14]. In particular, HDL targets the activation of eNOS via the phosphorylation at specific sites – Ser^1177^ (activation of the enzyme) and Thr^495^ (inhibition of the enzyme). In the present study a significant increase in HDL-mediated eNOS-Ser^1177^ phosphorylation was detected in the group assigned to the 10 months' lifestyle intervention program when compared to the usual care group. No change was documented at the eNOS-Thr^495^ residue. This result differs in some aspects to a recently published study by our group, documenting that lifestyle intervention as well as bariatric surgery did not affect HDL-mediated eNOS phosphorylation [16]. A possible explanation for this difference is that in the earlier study the patients remained obese (BMI-SDS above 2 and BMI percentile above 97%) even after 6 or 12 months of intervention, whereas the adolescents in the present study lost significant weight and can be classified as normal (BMI around 25 kg/m^2^; BMI-SDS around 1.1). This observation supports the hypothesis formulated in the previous study [16] that a certain threshold in body weight has to be reached by the intervention program in obese adolescents, before a significant effect on HDL function can be achieved. This is further supported by a study in overweight/obese women, where a diet intervention (6-month duration) did not alter eNOS activity [23]. In that study the intervention elicited only minor changes in body weight (minus 2.3%) and fat mass (minus 3%) when compared to the present one (body weight minus 31% and fat mass minus 41%).

One could wonder whether this HDL-induced change in eNOS phosphorylation is physiologically relevant. In an elegant study Teupe and colleagues documented that gene transfer of an eNOS construct, mimicking the phosphorylation of eNOS at Ser^1177^, resulted in an enhanced NO-mediated relaxation of bradykinin-stimulated porcine coronary arteries [24]. In the present study the change in HDL-mediated eNOS phosphorylation at Ser^1177^ and the change in endothelial function showed a positive correlation. Therefore, the HDL-mediated increase in eNOS-Ser^1177^ phosphorylation elicited by the intervention program may contribute to improvement of endothelial function in obese adolescents.

### 4.2. Lifestyle Intervention Program and Impact on HDL-Mediated Cholesterol Efflux

A key function of HDL is to promote reverse cholesterol transport from the periphery back to the liver. The critical step in this process is the cholesterol efflux from macrophages to HDL. This macrophage-specific cholesterol efflux capacity has been causally linked to atherosclerosis [25] and therefore an elevated cholesterol efflux capacity is inversely associated with the incidence of cardiovascular events [26]. Is a lifestyle intervention program capable of improving HDL-mediated cholesterol efflux capacity in obese individuals? In the present study the combination of restrictive diet and an exercise program over 10 months improved HDL-mediated efflux capacity significantly when compared to the usual care group (Figure 2). This finding is supported by 2 other studies [18, 23] where lifestyle intervention programs had a positive impact on cholesterol efflux capacity. It is worthwhile to note that in the study by Lesná et al. [18] a negative correlation was noted between the change in weight loss and the improvement in efflux capacity. The authors further concluded that it is necessary to decrease body weight for more than 4-5% to reach a biological significant increase in reverse cholesterol transport. The physiological relevance of the change in cholesterol efflux capacity is further supported by its positive correlation with the change in endothelial function.

### 4.3. Study Limitations

The results of the present study suggest that restoring HDL function, measured as HDL-induced eNOS phosphorylation and cholesterol efflux capacity, by lifestyle interventions improves endothelial function. This assumption is only based on correlation analysis but direct experimental evidence like HDL-induced vasodilation of aortic rings in an organ bath setting is missing. Therefore, modulation of other parameters like LDL, C reactive protein (CRP), or vessel stiffness by lifestyle intervention could also play an important role for improving vasomotor function. Improvement of inflammation by lifestyle intervention can be ruled out, since CRP does not change significantly, whereas LDL and vessel stiffness change significantly. Therefore, restoration of HDL function by lifestyle intervention may be only one out of several parameters potentially modulating endothelial function.

To gain molecular insights into lifestyle induced modulation of HDL function, a shotgun proteome approach would be helpful. In a recent study [16], analyzing the impact of a lifestyle intervention or Roux-en-Y gastric bypass on HDL function, no change in the overall signature was evident.

### 4.4. Future Perspectives

It would be interesting to determine if an improvement in exercise capacity or the significant weight loss is the driving force to improve HDL function. Since the present study only analyzed a small group of adolescents a study with a larger patient cohort is warranted. In addition the molecular mechanism(s) leading to functional impairment of HDL in obesity still remains unclear. Future studies have to clarify if differences in protein or lipid composition of HDL are a reason for impaired function or whether secondary protein modification, like carbonylation and methylation, is the key player.

## 5. Conclusion

In obese adolescents a stringent lifestyle intervention program results in enhanced reverse cholesterol transport and an HDL-mediated activation of eNOS. These effects may contribute to improvement of endothelial function. Nevertheless, to achieve these beneficial effects the lifestyle intervention program should be stringent enough to induce substantial weight loss.

## Figures and Tables

**Table 1:** Baseline characteristics and effects of the interventional program.

	Usual care group (*N* = 8)		Intervention group (*N* = 8)		*p* value baseline	*p* value intervention
	Baseline	10 mo	Baseline	10 mo		
Anthropometry						
Age (yrs)	15.1 ± 2.5	—	14.9 ± 3.6	—	0.88	—
Weight (kg)	114.6 ± 21.1	117.2 ± 22.6	109.1 ± 26.3	75.0 ± 10.7	0.88	**<0.001**
Weight, SDS	2.99 ± 0.75	2.93 ± 0.80	2.82 ± 0.91	1.07 ± 0.67	0.88	**<0.001**
BMI (kg/m^2^)	39.57 ± 6.21	39.79 ± 5.90	37.01 ± 5.98	25.03 ± 1.51	0.72	**<0.001**
BMI, SDS	3.00 ± 0.53	2.94 ± 0.51	2.82 ± 0.47	1.22 ± 0.22	0.72	**<0.001**
Body fat (%)	50.49 ± 5.79	50.38 ± 5.39	51.39 ± 2.10	30.61 ± 10.09	0.90	**<0.001**
Lean body mass (kg)	55.12 ± 14.27	56.22 ± 14.42	51.65 ± 14.05	50.19 ± 12.73	0.57	0.065
Fat free mass (kg)	58.42 ± 14.65	59.58 ± 14.85	54.69 ± 14.50	53.39 ± 13.40	0.51	**0.03**
Laboratory parameters						
hsCRP (mg/L)	0.63 ± 0.43	0.38 ± 0.27	0.33 ± 0.17	0.42 ± 0.46	0.51	0.57
Triglycerides (mg/dL)	89.00 ± 25.55	78.75 ± 15.19	106.75 ± 33.92	63.63 ± 27.90	0.28	**0.015**
Cholesterol (mg/dL)	143.1 ± 27.72	142.3 ± 20.27	155.4 ± 14.98	132.3 ± 25.01	0.51	0.083
HDL (mg/dL)	41.50 ± 3.01	39.75 ± 2.91	42.13 ± 4.04	48.50 ± 4.63	0.96	0.21
LDL (mg/dL)	80.61 ± 19.43	79.80 ± 15.50	88.15 ± 15.01	64.69 ± 12.25	0.442	**0.01**
Blood pressure/endothelial function						
systolic (mm Hg)	125.5 ± 3.6	122.3 ± 3.4	122.5 ± 2.9	109.2 ± 2.4	0.38	**0.015**
Diastolic (mm Hg)	64.7 ± 2.5	66.2 ± 2.5	60.5 ± 1.9	58.7 ± 1.7	0.51	0.13
Pulse wave velocity (m/s)	5.9 ± 0.7	6.9 ± 1.6	6.3 ± 1.2	5.2 ± 0.4	0.88	**0.021**
Max. dilation (AU)	1.58 ± 0.17	1.50 ± 0.11	1.37 ± 0.07	2.21 ± 0.35	0.69	0.072
Exercise/endothelial function						
VO_2 peak_/FFM (mL/min/kg)	40.69 ± 3.54	31.56 ± 1.06	39.15 ± 2.55	43.80 ± 3.24	0.28	**0.002**
Max. load (watt)	159.4 ± 44.3	160.0 ± 50.2	150.0 ± 38.5	196.7 ± 66.5	0.65	**0.002**
